# Evaluation of lidocaine as a dual-route prophylaxis in postoperative catheter-related bladder discomfort: a comprehensive systematic review and meta-analysis with trial sequential analysis and GRADE evaluation

**DOI:** 10.1007/s00210-025-04937-8

**Published:** 2026-01-14

**Authors:** Mohamed Abo Zeid, Kareem Khalefa, Mohamed Wagdy, Ahmad Alkheder, Michael Gerges, Hussein Kadhim Hussein, Hiba Jasim Hafedh, Rawaa M. Mohammed, Amr M. Abou Elezz

**Affiliations:** 1 Faculty of Medicine, Tanta University, Tanta, Egypt; 2 Faculty of Medicine, Modern University for Technology and Information, Cairo, Egypt; 3 Department of Otorhinolaryngology, Al-Mouwasat University Hospital, Damascus University, Damascus, Syria; 4 Faculty of Medicine, Damascus University, Damascus, Syria; 5 Faculty of Medicine, Syrian Private University, Damascus, Syria; 6 College of Nursing, University of Warith Al-Anbiyaa, Karbala, Iraq; 7 College of Nursing, University of Al-Qadisiyah, Al Diwaniyah, Iraq; 8 Nursing College, Al-Mustaqbal University, Hillah, Iraq

**Keywords:** Lidocaine, Catheter-related bladder discomfort, IV, Bladder irrigation

## Abstract

Catheter-related bladder discomfort (CRBD) is a common postoperative problem following urinary catheterization. Current pharmacologic options have limited use due to side effects. Lidocaine, with analgesic, anti-inflammatory, and antimuscarinic properties, may provide an effective and safer alternative. This study aims to systematically evaluate (1) the efficacy of intravenous (IV) lidocaine compared with normal saline, (2) the efficacy of intravesical lidocaine compared with normal saline, and (3) postoperative outcomes including opioid requirements, as specified in the PROSPERO registration (CRD420251155678). A systematic search of randomized controlled trials (RCTs) was conducted in accordance with PRISMA guidelines. Studies comparing intravenous or intravesical lidocaine with normal saline for CRBD prevention in adult surgical patients were included. Primary outcomes were CRBD incidence and severity at predefined postoperative time points. Secondary outcomes included opioid consumption when reported. Study quality was assessed using the RoB 2 tool, and the certainty of evidence was evaluated by GRADE and trial sequential analysis (TSA). Five RCTs, including 463 patients, were analyzed. Lidocaine significantly reduced moderate-to-severe CRBD compared with saline at 0 h (RR = 0.42, 95% CI 0.32–0.55), 1 h (RR 0.42, 95% CI 0.28, 0.61), and 2 h (RR 0.33, 95% CI 0.12, 0.90), but not at 6 h (RR 0.30, 95% CI 0.07, 1.22). No significant difference was observed in postoperative opioid consumption (MD − 0.56, 95% CI − 1.14, 0.03). TSA confirmed conclusive results at 0 and 1 h, with high-certainty evidence per GRADE. Both IV and transurethral lidocaine significantly reduce early postoperative CRBD. Lidocaine represents a safe, effective, and easily applicable option for short-term prevention of CRBD.

**Supplementary Information:**

The online version contains supplementary material available at 10.1007/s00210-025-04937-8.

## Introduction

Catheter-related bladder discomfort (CRBD) is a frequent postoperative complication observed in patients undergoing transurethral surgical procedures with an incidence varying between 47 and 90%. It is clinically characterized by an urge to void, a burning sensation originating in the suprapubic region and radiating toward the penis, and is frequently associated with significant discomfort (Bai et al. 2015; Agarwal et al. 2005).

CRBD can substantially intensify pain and provoke agitation, requiring immediate clinical management. CRBD is thought to result from catheter-induced irritation of the bladder mucosa and trigone, triggering afferent activation and involuntary detrusor contractions. Based on established bladder physiology and clinical response to antimuscarinic agents, these contractions are believed to be mediated predominantly through muscarinic M₃ receptors (Yamanishi et al. 2001; Markopoulos et al. 2025). Whether the irritation is caused by normal saline used in irrigation or by the catheter itself, it can trigger afferent nerve activity within the bladder. The detrusor muscle, responsible for bladder contraction during urination, is regulated by muscarinic acetylcholine receptors, with the M2 and M3 subtypes playing key roles. M3 receptors are directly involved in detrusor contraction and bladder emptying, while M2 receptors, though less prevalent, also contribute to bladder contractility and have been implicated in the development of CRBD and other bladder disorders. Activation of these receptors, particularly in response to mucosal irritation and catheter presence, can lead to involuntary bladder contractions and the hallmark symptoms of CRBD, including urgency, discomfort, and a persistent sensation of needing to void (Bai et al. 2015; Igawa 2000; Bozkurt and Sahin-Erdemli 2009).

Multiple drugs have been evaluated to manage CRBD, with varying degrees of clinical efficacy. These include antimuscarinic agents such as tolterodine, oxybutynin, solifenacin, and darifenacin; antiepileptics like gabapentin and pregabalin; analgesics including paracetamol, tramadol, and ketamine; as well as butylscopolamine and dexmedetomidine, but due to their side effects like dry mouth, sedation, nausea, and vomiting, this initiated finding novel methods like using local anesthetics such as lidocaine (Agarwal et al. 2005, 2007, 2006; Srivastava et al. 2015; Bala et al. 2012; Ergenoglu et al. 2012; Nam et al. 2015; Tauzin-Fin et al. 2007).

Lidocaine, administered intravenously (IV) or via bladder irrigation, mitigates CRBD by blocking sodium channels in the bladder mucosa, reducing afferent sensory signaling. This targeted mechanism offers a localized approach to symptom relief, potentially enhancing efficacy and minimizing systemic side effects compared to conventional treatments previously mentioned. In addition to its action locally on bladder mucosa, IV lidocaine infusion has analgesic and anti-inflammatory as well as antimuscarinic properties, but it is secondary and has a less significant effect. Thus, while a minimal antimuscarinic contribution cannot be excluded, the clinical benefit of lidocaine for CRBD is more credibly attributed to reduced afferent signaling and its systemic analgesic and anti-inflammatory effects (Marret et al. 2008; Lauretti 2008). IV lidocaine infusion therefore aligns with the underlying pathophysiology of CRBD and may contribute to reduced postoperative pain, nausea, and vomiting, supporting faster recovery and shorter hospital stay (Marret et al. 2008; Aguilar et al. 1980; Khan et al. 2016).

We conducted this study to evaluate the effectiveness of lidocaine administration in relieving CRBD after surgeries requiring catheter insertion.

## Methods

### Study design and registration

We meticulously conducted this systematic review and meta-analysis adhering to the PRISMA (Page et al. 2021)—Preferred Reporting Items for Systematic Reviews and Meta-Analyses—guidelines and following the Cochrane Collaboration’s recommendations. This study was registered in the International Prospective Register of Systematic Reviews (Prospero) under the registration number: CRD420251155678.

### Eligibility criteria

To establish our eligibility criteria, we adopted the PICO framework. We included randomized controlled trials (RCTs) that evaluated the efficacy of lidocaine (administered IV or via bladder irrigation) in preventing or reducing CRBD in surgical patients requiring postoperative urinary catheter insertion. Eligible comparators included placebo or normal saline. Outcomes of interest were the incidence or severity of moderate to severe CRBD and perioperative opioid consumption (e.g., tramadol, morphine, and fentanyl). We excluded case reports, animal studies, reviews, editorials, studies with only abstracts or unavailable full text, studies with overlapping or duplicate data, and studies involving other pharmacological or non-pharmacological interventions without a relevant comparison group. Detailed eligibility criteria of the included studies are provided in Supplementary Table 1.

### Search strategy

A comprehensive search of electronic databases (PubMed, Embase, Cochrane Library, and Scopus) was conducted from database inception to 19th September 2025. The following search strategy was used in PubMed and adapted for other databases: (“Lidocaine” OR “Lignocaine” OR “i.v. lidocaine” OR “Xylocaine” OR “bladder instillation” OR “intravenous lidocaine” OR “IV lidocaine” OR “intravesical lidocaine” OR “bladder irrigation”) AND (“catheter related bladder discomfort” OR “catheter-related bladder discomfort” OR “CRBD” OR “bladder pain” OR “catheter pain” OR “catheter-related pain” OR “catheter-related urinary discomfort” OR “catheter-associated urinary discomfort”). The reference lists of included articles and relevant reviews were also screened to identify additional eligible studies. No language restrictions were applied.

### Study selection and data extraction

Two reviewers independently screened titles and abstracts using Rayyan website (Ouzzani et al. 2016), followed by full-text review for eligibility. Disagreements were resolved by discussion or consultation with a third reviewer. Data extracted included study characteristics (author, year, country, and study design), patient population, type of surgery, intervention details (dose, route, timing of lidocaine administration), comparator, and outcome measures.

### Quality assessment

To assess the methodological quality of the included RCTs, we used the Cochrane Risk of Bias 2.0 tool (Sterne et al. 2019). The tool evaluates six key aspects: (1) bias in random sequence generation, (2) bias due to deviations from intended interventions, (3) bias due to missing outcome data, (4) bias in outcome measurement, (5) bias in the selection of reported results, and (6) overall bias. Each aspect is classified as “low risk,” “some concern,” or “high risk.” The GRADE approach was used to assess the strength of the evidence (Schünemann et al. n.d.).

### Data synthesis

Where possible, data were pooled using a random-effects meta-analysis. Dichotomous outcomes (e.g., incidence of moderate to severe CRBD) were expressed as risk ratios (RRs) with 95% confidence intervals (CIs). Continuous outcomes (e.g., opioid consumption) were summarized as mean differences (MDs) or standardized mean differences (SMDs). CRBD was assessed at four postoperative time points: immediately upon arrival to the recovery area (0 h), and subsequently at 1, 2, and 6 h postoperatively. Statistical heterogeneity was assessed using the Chi^2^ test and the *I*^2^ statistic. Pre-specified subgroup analyses were planned according to the route of lidocaine administration (IV vs intravesical) and follow-up time points.

### Trial sequential analysis

To ensure the conclusiveness of our findings and minimize the risk of false positives (Type I errors), we applied a trial sequential analysis (TSA) utilizing the Copen-Hagen TSA program version 0.9.5.10 Beta (Thorlund et al. 2017). We employed parameters including a type 1 error (*α*) of 5%, a type 2 error (*β*) of 20% corresponding to a statistical power of 80%. We employed a model variance-based heterogeneity correction. For each outcome of interest, we generated both a monitoring boundaries plot and a penalized *z*-curve plot, ensuring the robustness of our meta-analytic findings regarding our primary outcome. As for the monitoring boundaries plot, it employed a superiority boundary based on the O’Brien–Fleming alpha spending function to mitigate type 1 errors and a futility boundary based on the O’Brien–Fleming beta spending function to mitigate type 2 errors. As for the penalized *z*-curve plot, the cumulative *z* statistic was penalized by applying the Law of Iterated Logarithm incorporating a *λ* value of 2. Both plots included a required information size (RIS) axis to determine whether the data is sufficient or not.

## Results

### Search results and study selection

**Fig. 1:**
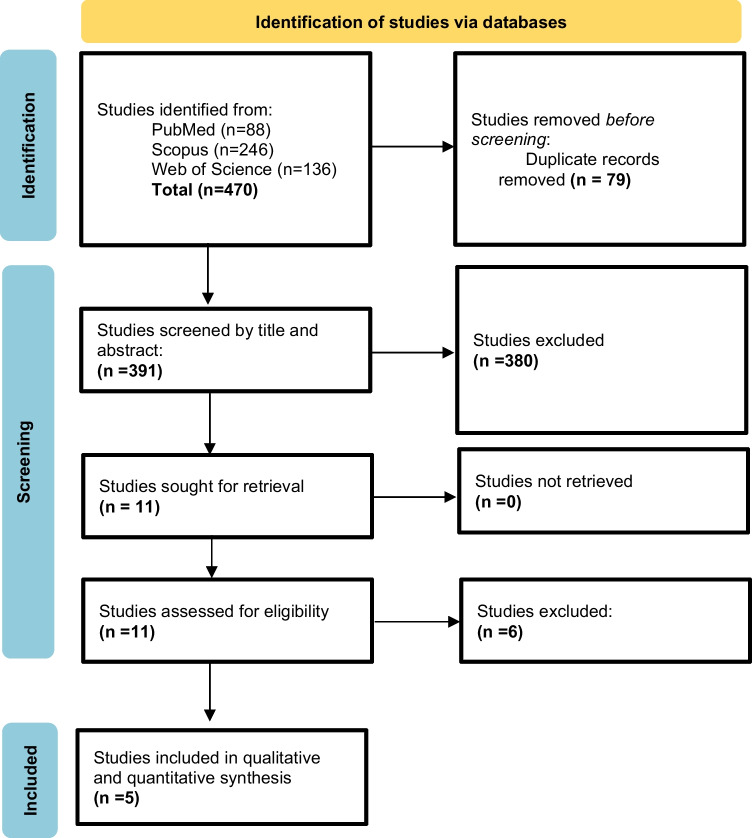
PRISMA flow chart

On conducting a systematic search encompassing PubMed, Scopus, and Web of Science, 470 records were retrieved. After exclusion of 79 duplicates, the remaining 391 records were screened for their titles and abstracts, followed by full-text screening; 380 records did not meet our eligibility criteria. Finally, five eligible full-text articles were included in the final analysis (Fig. 1).

### Characteristics of the included studies

A total of five RCTs were included in this study. Among these, three studies evaluated the effect of IV lidocaine, while two applied transurethral lidocaine. In all trials, normal saline was employed as the control. Participants in three RCTs underwent urological procedures, including transurethral resection of bladder tumors (TURBT), ureterorenoscopic lithotripsy (URSL), and transurethral resection of the prostate (TURP). The remaining two trials included non-urological surgeries; one RCT included patients undergoing hysterectomy, and the other included lumber spine surgery (Table 1).

**Table 1:** Summary of included studies

Study ID (Country)	Study Design	Study arms			Type of surgery	Inflated balloon and volume protocol	Catheter size
		Intervention		Control			
		Intervention route	Protocol				
Chantrapannik et al. (2024) (India)	RCT	IV Lidocaine	1.5 mg/kg followed by 2 mg/kg/h	NS	• Complex fusion lumbar spinal surgery involving at least two levels.	Balloon was inflated with 10 ml of sterile water (secured to the thigh with plaster).	≥ 20 Fr.
Kim et al. 2020 (Korea)	RCT	IV Lidocaine	1.5 mg/kg followed by 2 mg/kg/h	NS	• Transurethral Resection of Bladder Tumors (TURBT)	Balloon was inflated with 10 mL of distilled water. (A 2% lidocaine gel was used to lubricate the catheter).	≥ 20 Fr.
Li et al. 2019 (China)	RCT	IV Lidocaine	2 mg/kg followed by 1.5 mg/kg/h	NS	• Elective open abdominal hysterectomy or hysteromyomectomy	Balloon was inflated with 10mL normal saline.	16 Fr.
Lin et al. (2024) (Taiwan)	RCT	Transurethral 0.05% Lidocaine	500 mg in 1 L NS bladder irrigation	NS	• Elective transurethral surgery under general anesthesia • Ureterorenoscopic lithotripsy (URSL) • Transurethral resection of the prostate (TURP)	-	22 Fr.
Singh et al. 2023 (Thailand)	RCT	Transurethral 0.01% Lidocaine	100 mg in 1 L NS bladder irrigation	NS	• Transurethral Resection of Bladder Tumors (TURBT)	Balloon was inflated with 10 mL. (A 2% lidocaine gel was used to lubricate the catheter).	≥ 20 Fr.

*RCT*; Randomized controlled trial, *NS*; Normal Saline, *TURBT*; Transurethral Resection of Bladder Tumors, *URSL*; Ureterorenoscopic lithotripsy, *TURP*; Transurethral resection of the prostate, *Fr*; French

Overall, 463 patients were enrolled across the included studies, with 231 receiving lidocaine and 232 receiving normal saline. The mean age of participants ranged from 44.4 ± 6.1 to 66.2 ± 10.61 years. Common comorbidities included diabetes mellitus and hypertension. The mean operative time varied notably across the included studies, ranging from 63.33 ± 15.16 to 320.6 ± 86.1 min (Table 2).

**Table 2:** Baseline characteristics and demographics of included studies

Study ID	Study arms	Study participants	Age (years), mean (SD)	BMI (kg/m^2^), mean (SD)	Gender (males) *N* (%)	ASA classifications			Operative time, mean (SD)	DM	Hypertension
						**1**	**2**	**3**			
Chantrapannik et al. (2024)	**Lidocaine**	40	60.2 (11.2)	25.4 (3.8)	Males	12 (30.0)	28 (70)	NA	296.4 (98.7)	14 (35.0%)	23 (57.5%)
	**Normal saline**	40	58.0 (11.2)	26.2 (4.0)		13 (32.5)	27 (67.5)	NA	320.6 (86.1)	8 (20.0%)	23 (57.5%)
Kim et al. (2020)	**Lidocaine**	66	64.33 (12.13)	24.6 (3.0)	Males	28 (42.4)	38 (57.6	NA	66.67 ± 18.95	NA	NA
	**Normal saline**	66	66.17 (10.61)	24.6 (3.3)		17 (25.8)	49 (74.2)	NA	63.33 ± 15.16	NA	NA
Li et al. 2019)	**Lidocaine**	39	45.0 (6.4)	NA	NA	NA	NA	NA	124.1 ± 31.5	NA	NA
	**Normal saline**	39	44.4 (6.1)	NA	NA	NA	NA	NA	119.5 ± 28.6	NA	NA
Lin et al. (2024)	**Lidocaine**	39	63.9 (6.6)	24.8 (2.9)	NA	NA	27	12	NA	9 (23.1%)	17 (43.6%)
	**Normal saline**	40	63.5 (6.9)	25.5 (3.7)	NA	NA	31	9	NA	7 (17.5%)	9 (22.5%)
Singh et al. (2023)	**Lidocaine**	47	59.34 (11.05)	25.3 (1.75)	36 (76.6)	39 (83.0)	8 (17.0)	NA	94.87 ± 8.73	3 (6.4%)	4 (8.5)
	**Normal saline**	47	54.4 (14.13)	26.1 (2.44)	35 (74.5)	37 (78.8)	10 (21.3)	NA	91.97 ± 11.52	2 (4.3%)	5 (10.6)

### Quality assessment

**Fig. 2:**
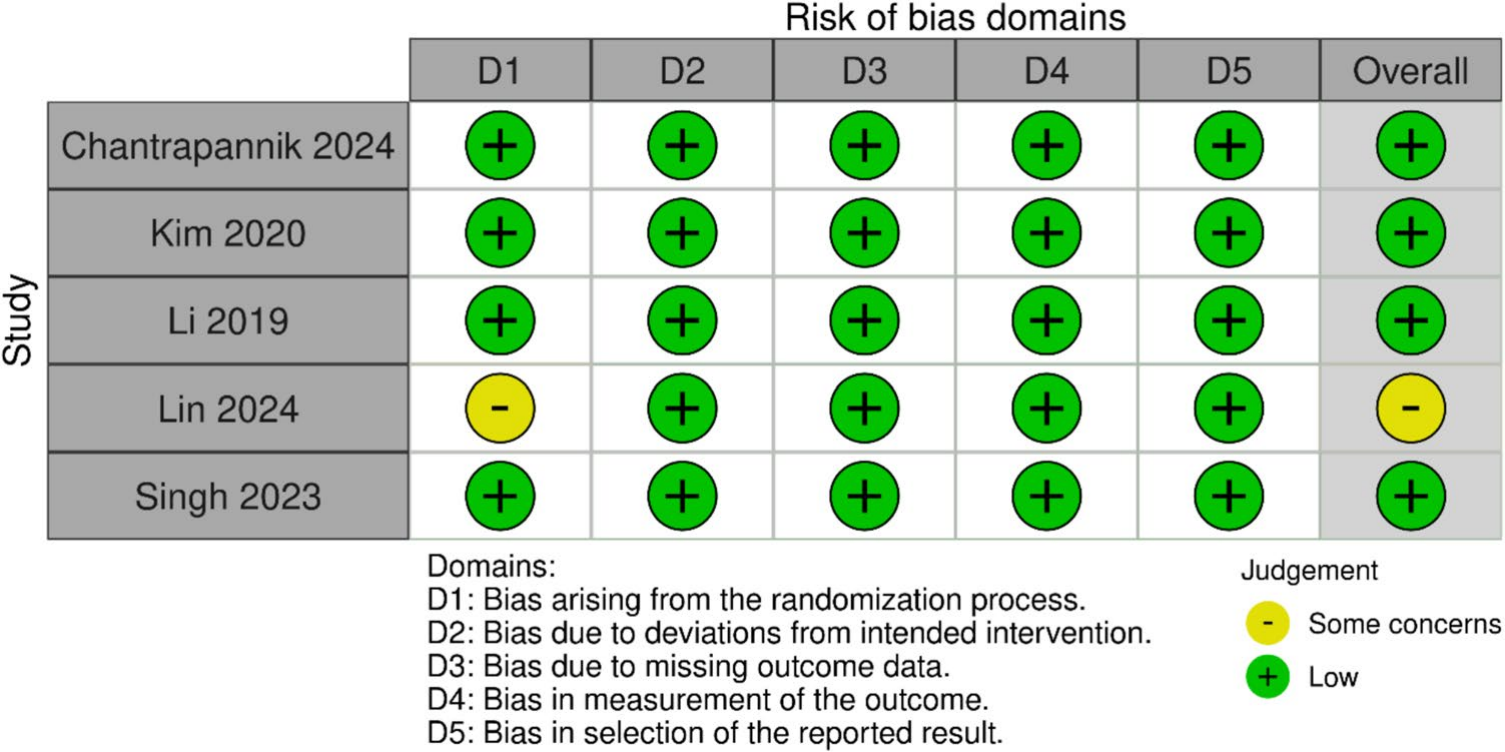
Risk of bias assessment of the included studies

The risk of bias assessed using the Risk of Bias 2 (ROB-2) tool. Only one study showed some concerns regarding the randomization process, whereas the other four showed low risk in all domains (Fig. 2).

### Efficacy outcomes

#### Incidence of moderate to severe CRBD comparing lidocaine and normal saline (including both IV lidocaine and bladder irrigation)

**Fig. 3:**
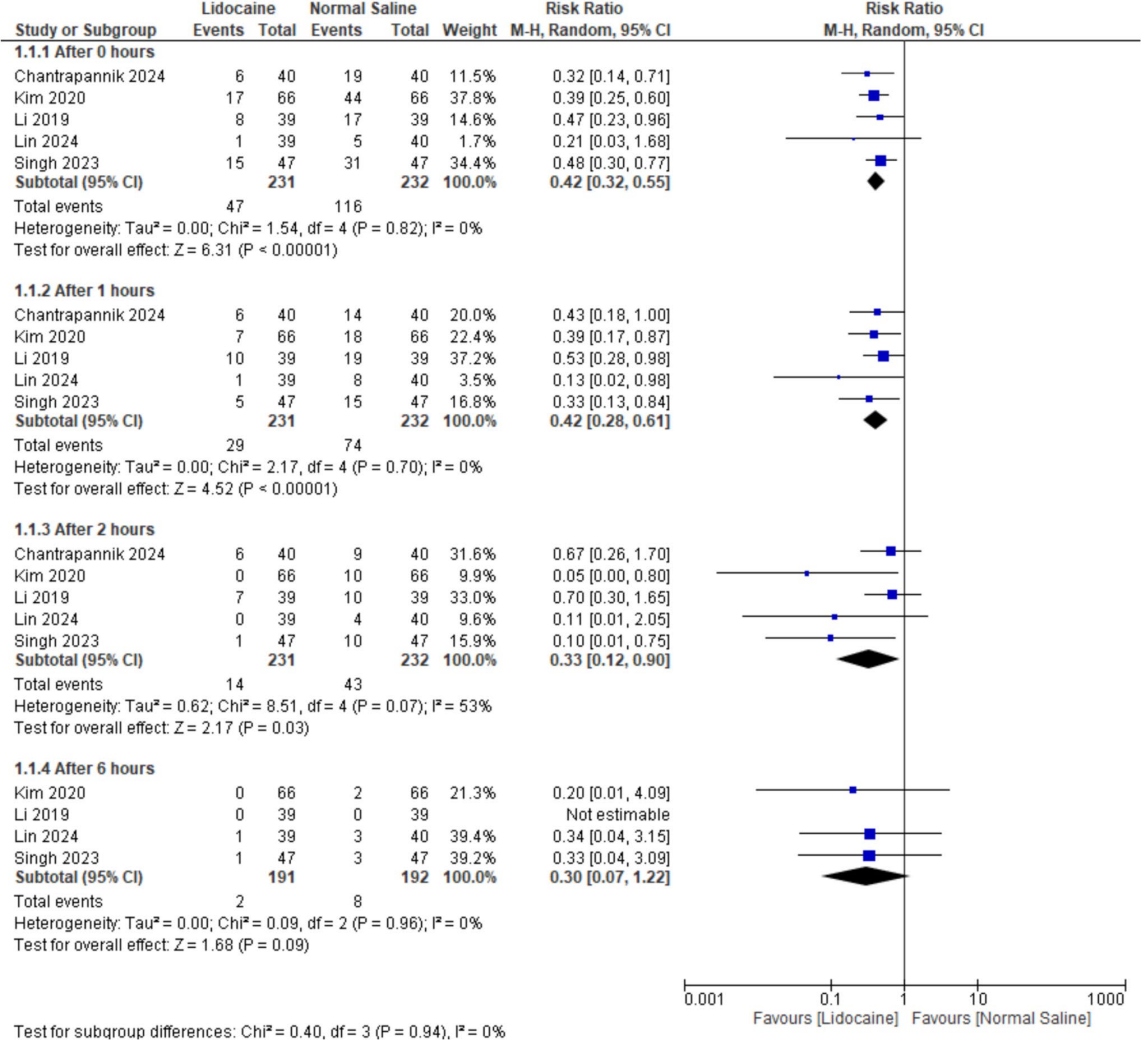
Forest plot for the incidence of moderate to severe CRBD comparing lidocaine and normal saline (including both IV lidocaine and bladder irrigation)

The pooled analysis revealed a statistically significant reduction in the incidence of moderate to severe CRBD favoring lidocaine compared to normal saline at 0 h, 1 h, and 2 h postoperatively (RR = 0.42, 95% CI [0.32 to 0.55], *P* < 0.00001), (RR = 0.42, 95% CI [0.28 to 0.61], *P* < 0.00001), and (RR = 0.33, 95% CI [0.12 to 0.90], *P* = 0.03), respectively. Moderate heterogeneity was observed in the 2-h subgroup (*P* = 0.07, *I*^2^ = 53%). However, analysis after 6 h demonstrated a non-significant difference favoring either of the two groups (RR = 0.30, 95% CI [0.07 to 1.22], *P* = 0.09), with no heterogeneity between subgroups (Fig. 3).

#### Incidence of moderate to severe CRBD comparing lidocaine and normal saline (including only IV lidocaine)

**Fig. 4:**
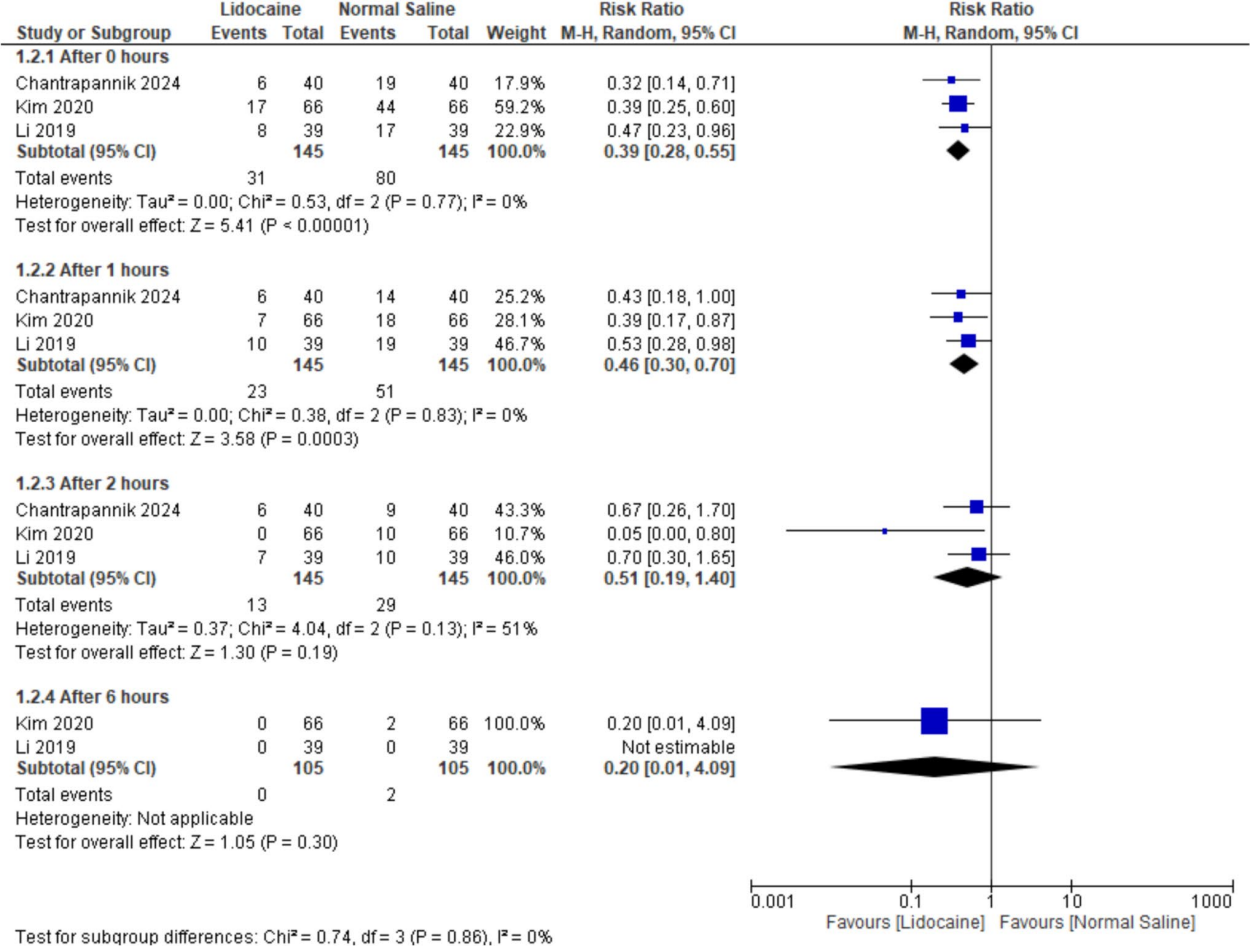
Forest plot for the incidence of moderate to severe CRBD comparing lidocaine and normal saline (including only IV lidocaine)

Regarding subgroup analysis restricted to IV lidocaine, results revealed statistically significant differences favoring IV lidocaine over normal saline at 0 h and 1 h postoperatively (RR = 0.39, 95% CI [0.28 to 0.55], *P* < 0.00001), (RR = 0.46, 95% CI [0.30 to 0.70], *P* = 0.0003) with no heterogeneity in either subgroup. However, results at 2 h and 6 h showed a non-significant difference favoring either of the two groups (RR = 0.51, 95% CI [0.19 to 1.40], *P* = 0.19),(RR = 0.20, 95% CI [0.01 to 4.09], *P* = 0.30), with moderate heterogeneity in the 2-h subgroup (*P* = 0.13, *I*^2^ = 51%) (Fig. 4).

#### Incidence of moderate to severe CRBD comparing lidocaine and normal saline (including only lidocaine bladder irrigation)

**Fig. 5:**
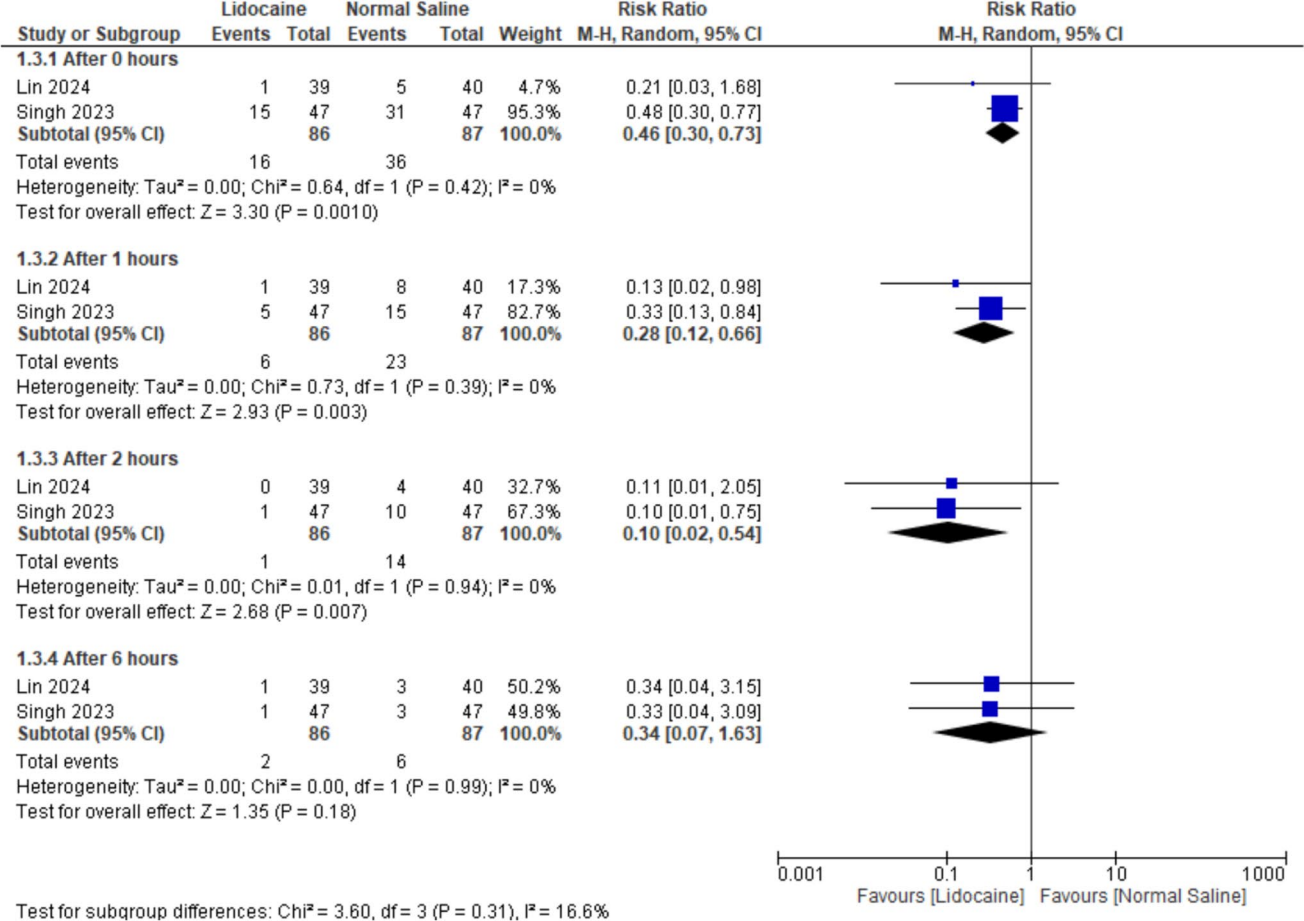
Forest plot for the incidence of moderate to severe CRBD comparing lidocaine and normal saline (including only lidocaine bladder irrigation)

Similarly, when analyzing studies utilizing only lidocaine via bladder irrigation, results showed statistically significant favoring lidocaine over normal saline at 0 h, 1 h, and 2 h postoperatively (RR = 0.46, 95% CI [0.30 to 0.73], *P* = 0.0010), (RR = 0.28, 95% CI [0.12 to 0.66], *P* = 0.003), and (RR = 0.10, 95% CI [0.02 to 0.54], *P* = 0.007) with no heterogeneity in any subgroup. However, results at 6 h indicated a non-significant difference favoring either of the two groups (RR = 0.34, 95% CI [0.07 to 1.63], *P* = 0.18) with no heterogeneity (*P* = 0.99, *I*^2^ = 0%). And no heterogeneity between subgroups (Fig. 5).

#### Postoperative opioid requirements comparing lidocaine and normal saline

**Fig. 6:**
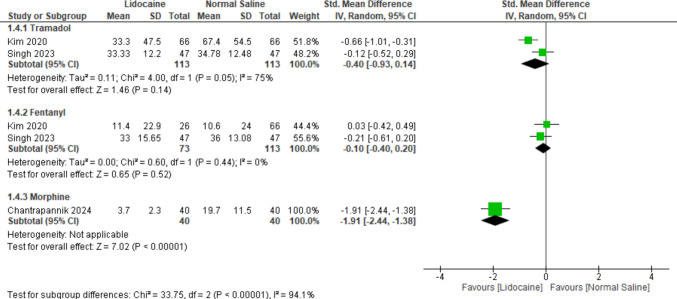
Forest plot comparing mean differences (MDs) of postoperative opioid requirements comparing lidocaine and normal saline

Regarding postoperative opioid consumption, subgroup analysis by opioid type revealed no significant differences in the tramadol and fentanyl subgroups (MD = − 0.40, 95% CI [− 0.93 to 0.14], *P* = 0.14), (MD = − 0.10, 95% CI [− 0.40 to 0.20], *P* = 0.52), respectively, with substantial heterogeneity (*P* = 0.05, *I*^2^ = 75%) in the tramadol subgroup. In contrast, the morphine subgroup analysis demonstrated a significant reduction favoring lidocaine over normal saline (MD = − 1.91, 95% CI [− 2.44 to − 1.38], *P* < 0.00001) with substantial heterogeneity between subgroups (*P* = 0.00001, *I*^2^ = 94.1%); however, this analysis included a single study, so it should be interpreted cautiously (Fig. 6).

### Trial sequential analysis (TSA)

TSA on risk ratios (RR) of CRBD at 0 h comparing lidocaine to normal saline showed that the required information size of 218 was reached. Moreover, the final point on the cumulative *z*-curve passed the superiority boundary (true positive region), indicating a conclusive result (Supplementary Fig. 1 A). Moreover, the penalized *z*-curve passed the conventional boundary of (*z* = 1.96) (Supplementary Fig. 1B).

Similarly, TSA of CRBD after 1 h showed that the required information size of 116 was reached. Moreover, the final point on the cumulative *z*-curve passed the superiority boundary (true positive region), indicating a conclusive result (Supplementary Fig. 2 A). Similarly, the penalized *z*-curve passed the conventional boundary of (*z* = 1.96) (Supplementary Fig. 2B).

Regarding TSA of CRBD, after 2 h, the cumulative *z*-curve passed the conventional boundary but did not pass the superiority boundary (false positive region), indicating a non-conclusive result (Supplementary Fig. 3A). Although the penalized *z*-curve passed the conventional boundary of (*z* = 1.96) (Supplementary Fig. 4B), the required information size of 765 was not reached.

In contrast, TSA regarding CRBD after 6 h demonstrated that the cumulative *z*-curve did not pass the conventional boundary nor the superiority boundary (false negative region), indicating a non-conclusive result (Supplementary Fig. 4 A). Moreover, the penalized *z*-curve did not pass the conventional boundary of (*z* = 1.96) (Supplementary Fig. 4B). The required information size of 791 was not reached.

### Subgrouping analysis and leave-one-out sensitivity analysis

Our subgroup analysis, stratified by postoperative time points (0, 1, 2, 6) and lidocaine administration route (IV, bladder irrigation), confirmed the efficacy of lidocaine in the reduction of incidence of moderate to severe CRBD compared to normal saline. No significant differences were observed between compared subgroups, and statistical heterogeneity was minimal (Table 3).

**Table 3:** Summary of findings with comparative subgroup analysis of CRBD incidence: lidocaine vs normal saline

Analysis description	No. of studies (No. of patients)	CRBD risk ratio [± 95% CI]	Heterogeneity, *I*^2^ (%)	*P*-value	*P-*value subgroup comparison
*Risk of moderate to severe CRBD after 0 h*					
Overall	5 (463)	0.42 [0.32 to 0.55] **⨁⨁⨁⨁**	*I*^2^ = 0%	*P* < 0.00001*	-
IV lidocaine subgroup	3 (290)	0.39 [0.28 to 0.55]	*I*^2^ = 0%	*P* < 0.00001*	*P* = 0.54
Lidocaine irrigation subgroup	2 (173)	0.46 [0.30 to 0.73]	*I*^2^ = 0%	*P* = 0.001*	
*Risk of Moderate to severe CRBD After 1 h*					
Overall	5 (463)	0.42 [0.28 to 0.61] **⨁⨁⨁⨁**	*I*^2^ = 0%	*P* < 0.00001*	-
IV lidocaine subgroup	3 (290)	0.46 [0.30 to 0.70]	*I*^2^ = 0%	*P* = 0.0003*	*P* = 0.31
Lidocaine irrigation subgroup	2 (173)	0.28 [0.12 to 0.66]	*I*^2^ = 0%	*P* = 0.003*	
*Risk of moderate to severe CRBD after 2 h*					
Overall	5 (463)	0.33 [0.12 to 0.90] **⨁⨁◯◯**	*I*^2^ = 53%	*P* = 0.03*	-
IV lidocaine subgroup	3 (290)	0.51 [0.19 to 1.40]	*I*^2^ = 51%	*P* = 0.19	*P* = 0.11
Lidocaine irrigation subgroup	2 (173)	0.10 [0.02 to 0.54]	*I*^2^ = 0%	*P* = 0.007*	
*Risk of moderate to severe CRBD after 6 h*					
Overall	4 (383)	0.30 [0.07 to 1.22] **⨁⨁⨁◯**	*I*^2^ = 0%	*P* = 0.09	-
IV lidocaine subgroup	2 (210)	0.20 [0.01 to 4.09]	-	*P* = 0.30	*P* = 0.76
Lidocaine irrigation subgroup	2 (173)	0.34 [0.07 to 1.63]	*I*^2^ = 0%	*P* = 0.18	

CRBD; catheter–related bladder discomfort

To ensure the robustness of our evidence, we conducted a leave-one-out sensitivity analysis in multiple scenarios, excluding one study in each scenario to make sure that the overall effect estimates were not dependent on a single study. Our findings demonstrated notable stability, as the pooled significance regarding the incidence of moderate to severe CRBD comparing lidocaine and normal saline remained unaffected across all follow-up time points except at 2 h postoperative, where the exclusion of either Lin et al.’s (2024), Singh et al.’s (2023), or Kim et al.’s (2020) resulted in the loss of statistical significance (Supplementary Figs. 5–8).

### Publication bias

Generally, funnel plots demonstrated a notable degree of symmetry, suggesting a low risk of publication bias. However, Egger’s test was not performed due to the small number of studies included (Supplementary Fig. 9).

### GRADE evaluation

Grade results and summary comparative analysis are demonstrated in (Table 3). The quality of evidence regarding the incidence of moderate to severe CRBD comparing lidocaine and normal saline ranged from low to high level of confidence across different time points. Downgrades in evidence quality were mainly attributed to inconsistency and imprecision. A detailed GRADE evaluation is provided in Supplementary Table 2.

## Discussion

This systematic review and meta-analysis, incorporating data from five RCTs and 463 patients, provides a comprehensive evaluation of the efficacy of lidocaine for the prevention of CRBD. The principal findings indicate that lidocaine administration, whether IV or via bladder irrigation, significantly reduces the incidence of moderate to severe CRBD in the immediate postoperative period (0–2 h), with a particularly pronounced and sustained effect observed for the intravesical route. However, this benefit appears to be attenuated by the 6-h mark. Furthermore, while the overall effect on postoperative opioid consumption was not statistically significant, a notable, significant reduction in morphine requirements was identified in a specific subgroup.

The pathophysiological mechanism of CRBD is primarily mediated through the activation of muscarinic receptors, specifically M2 and M3 subtypes, in the bladder wall, leading to involuntary detrusor contractions and the characteristic symptoms of urgency and suprapubic discomfort (Yamanishi et al. 2001; Markopoulos et al. 2025; Chantrapannik et al. 2024). Lidocaine’s efficacy can be attributed to its dual mechanisms of action. Locally, via intravesical instillation, it acts as a sodium channel blocker on the bladder mucosa, dampening afferent sensory signaling directly at the source (Lin et al. 2024). Systemically, IV lidocaine is proposed to exert analgesic, anti-inflammatory, and antimuscarinic properties, which collectively contribute to reducing bladder overactivity and the associated discomfort (Chantrapannik et al. 2024). When considering systemic (IV) lidocaine, its safety profile requires careful attention. An international consensus statement highlights it as a “high-risk” medicine due to its narrow therapeutic index and potential for serious toxicity, which is influenced by dosage, infusion rate, patient comorbidities, and concurrent local anesthetic use. Recommended safe practice for postoperative infusion includes a loading dose not exceeding 1.5 mg/kg ideal body weight over 10 min, followed by an infusion of ≤ 1.5 mg/kg/h for typically no longer than 24 h, with close monitoring and avoidance of concurrent neural blockade. These guidelines underscore that the decision to use IV lidocaine must weigh potential benefits against these risks, tailored to the individual patient and surgical procedure (Foo et al. 2021). The significant risk reduction (RR = 0.42 at 0 h) observed in our primary analysis strongly supports the biological plausibility of these mechanisms in mitigating CRBD.

A critical finding of our subgroup analysis is the differential efficacy based on the route of administration. Intravesical lidocaine demonstrated a robust and statistically significant reduction in moderate to severe CRBD at 0, 1, and 2 h postoperatively. For instance, the risk ratio at 2 h was 0.10, indicating a 90% reduction in risk compared to normal saline, with no heterogeneity. This suggests a potent and consistent local effect. The direct application of lidocaine to the urothelium ensures a high local concentration, providing targeted relief with minimal systemic exposure.

In contrast, the efficacy of IV lidocaine, while significant at 0 and 1 h, was not sustained at 2 and 6 h. This temporal pattern may reflect the pharmacokinetic profile of IV lidocaine, where declining plasma concentrations after the cessation of infusion might lead to a reduction in its therapeutic effect on the bladder (Chantrapannik et al. 2024). These findings position IV lidocaine as a valuable option for managing early CRBD, particularly in surgeries where bladder irrigation is not feasible or when systemic analgesic and opioid-sparing effects are also desired.

The analysis of postoperative opioid consumption revealed intriguing insights. The overall pooled estimate did not reach statistical significance, which can be attributed to significant heterogeneity and the varying potency of the opioids analyzed (tramadol, fentanyl, morphine). However, in the morphine subgroup, lidocaine administration resulted in a substantial and significant reduction in requirements (MD = − 1.91 mg). This finding is clinically relevant, as morphine is a potent opioid, and any reduction can potentially lower the incidence of dose-related adverse effects such as nausea, vomiting, and respiratory depression. This opioid-sparing effect aligns with the known systemic analgesic properties of IV lidocaine, as demonstrated in complex spine surgery where it significantly reduced 24-h morphine consumption (Chantrapannik et al. 2024).

When contextualizing our findings within the broader therapeutic landscape for CRBD, it is important to compare lidocaine to other interventions. A network meta-analysis by Ren et al. (2023) evaluated 16 different interventions and found nefopam to be the most effective in reducing CRBD incidence and severity (Ren et al. 2023). Another systematic review by Li et al. (2022) listed multiple effective drugs, including dexmedetomidine, gabapentin, tolterodine, and ketamine (Li et al. 2022). Lidocaine offers a distinct advantage over many of these systemic agents due to its favorable side effect profile. Antimuscarinic drugs like tolterodine are associated with dry mouth, constipation, and blurred vision (Agarwal et al. 2005), while gabapentin and pregabalin can cause dizziness and sedation. The included studies in our analysis reported no serious adverse events related to lidocaine, with only minor side effects such as drowsiness reported at similar rates between lidocaine and control groups. This safety profile makes lidocaine, particularly the intravesical route, an attractive alternative for patients who may be intolerant to the side effects of other medications.

Our findings on lidocaine’s efficacy should be interpreted within the broader context of available interventions for CRBD. Network meta-analyses (NMAs) have attempted to rank multiple treatments. One NMA found nefopam to be most effective in reducing both the incidence and severity of CRBD at 1-h postoperatively (Thorlund et al. 2017). Conversely, an earlier NMA concluded that gabapentin 1200 mg was best for reducing incidence, while tolterodine was superior for reducing severity (Hur et al. 2019). While these NMAs provide valuable hierarchies, direct comparisons with lidocaine are absent. Our analysis demonstrates that both intravenous and intravesical lidocaine are significantly effective in the critical early postoperative period (0–2 h), offering a favorable alternative. Lidocaine’s distinct mechanism of action and potentially preferable side-effect profile, especially for intravesical administration, position it as a compelling option, particularly when systemic side effects of other ranked drugs are a concern.

The clinical implications of our findings are substantial. For surgical procedures involving significant bladder manipulation, such as transurethral resections (TURBT, TURP), the use of intravesical lidocaine irrigation can be seamlessly integrated into the postoperative protocol. It provides a targeted, non-systemic, and effective means of preventing one of the most common and distressing postoperative complaints. For other major surgeries, like complex spinal procedures, where postoperative pain and CRBD are both significant concerns, IV lidocaine infusion presents a multi-modal option that addresses both issues simultaneously, while also reducing potent opioid requirements (Chantrapannik et al. 2024).

The high baseline incidence of CRBD, particularly after urological procedures like TURBT, underscores a significant unmet clinical need. Our findings demonstrate that lidocaine effectively addresses this issue, with intravesical administration showing a remarkably sustained reduction in discomfort up to 2 h postoperatively. This is clinically vital as CRBD peaks during this immediate recovery phase, often triggering agitation and complicating recovery. Utilizing a targeted, local intervention during this critical window can significantly enhance early patient comfort and potentially reduce behavioral responses that risk surgical site integrity (Bai et al. 2015).

Identifying patients most likely to benefit from prophylaxis is crucial for optimizing resource use. Previous research has established that factors such as younger age (< 50 years), male gender, and specific surgeries like gynecological procedures significantly increase the risk of moderate-to-severe CRBD (Lim and Yoon 2017). Our results, which show pronounced efficacy of lidocaine, suggest that it is an excellent candidate for targeted prophylaxis in these high-risk populations. Implementing a risk-stratified approach could improve overall management efficiency and patient satisfaction in the postoperative period.

When considering the therapeutic landscape, the favorable side-effect profile of lidocaine is a key advantage. While drugs like gabapentin are effective, their use can be limited by dose-related side effects such as dizziness and sedation, especially at higher doses needed for efficacy (Bala et al. 2012). Lidocaine, particularly via the intravesical route, offers potent relief without these systemic burdens, making it a suitable option for a broader patient cohort, including those who may be intolerant to the adverse effects of oral systemic agents.

Several limitations of this review warrant consideration. First, the number of included studies is modest (*n* = 5), which limits the power of some subgroup analyses and generalizability. Second, there was notable heterogeneity in the analysis of opioid consumption and the 2-h CRBD outcome for IV lidocaine, likely stemming from differences in surgical types, anesthetic techniques, and opioid administration protocols. Third, the follow-up period in the included trials was generally short, preventing any assessment of lidocaine’s effect on long-term outcomes such as patient satisfaction beyond 24 h or hospital length of stay. Consequently, the impact of effective early CRBD relief on these important patient-centered and health-economic endpoints remains an important area for future research. Fourth, as with any meta-analysis, the quality of our conclusions is dependent on the quality of the primary studies; although the risk of bias was generally low, some concerns were present in one study. Finally, the optimal dosing and concentration for intravesical lidocaine remain to be fully elucidated, with studies using concentrations ranging from 0.01% to 0.05% (Chantrapannik et al. 2024; Lin et al. 2024). Fifth, while our analysis confirms the overall efficacy of lidocaine, the aggregated nature of the data precluded subgroup analyses to investigate the influence of potential risk factors—such as patient age, gender, or specific comorbidities—on treatment response. Consequently, the efficacy of lidocaine within distinct high-risk populations could not be evaluated. Furthermore, the included trials did not provide standardized or reportable data on adverse events related to lidocaine administration. Consequently, our review could not assess the tolerability and safety profile of lidocaine in this specific context, which is a critical consideration given its narrow therapeutic window.

This meta-analysis provides robust evidence that lidocaine is an effective intervention for reducing the incidence and severity of early postoperative CRBD. Intravesical lidocaine irrigation offers a particularly potent and targeted effect, while IV lidocaine provides a valuable systemic option with additional analgesic benefits. Given its efficacy and favorable safety profile, lidocaine should be considered a first-line prophylactic option within a multimodal strategy for managing CRBD, especially in urological procedures. Future research should focus on large-scale RCTs to directly compare the efficacy of different lidocaine administration routes and to establish standardized dosing regimens for intravesical instillation.

## Future research directions

To address the limitations identified and build upon the promising findings of this analysis, several key avenues for future research are warranted. First, large-scale, multi-center randomized controlled trials with adequate power are essential to confirm the efficacy of both intravenous and intravesical lidocaine, particularly for outcomes where current evidence is limited or heterogeneous, such as opioid-sparing effects and CRBD relief beyond 2 h for the IV route. Second, these trials should aim to establish optimal and standardized dosing regimens for intravesical lidocaine instillation, as concentrations in existing studies vary considerably. Third, future studies should be designed to permit robust subgroup analyses based on known risk factors (e.g., age, gender, type of surgery) to better define which patient populations derive the greatest benefit from lidocaine prophylaxis, enabling a more personalized approach. Fourth, extending the follow-up period to assess the impact of effective early CRBD management on patient-centered outcomes—such as overall satisfaction, quality of recovery scores, and hospital length of stay—would provide valuable insights into its broader clinical and economic utility. Fifth, investigating lidocaine within a multimodal analgesia framework is crucial. Future trials should evaluate its potential synergistic effects when combined with other non-opioid analgesics (e.g., gabapentinoids, NSAIDs, or acetaminophen) or as part of a structured opioid-sparing regimen, to determine whether combination therapy enhances CRBD prevention, improves overall pain control, and further reduces opioid-related adverse events. Finally, prospective studies should systematically document and report lidocaine-related adverse events to thoroughly establish its safety profile in the context of CRBD prevention.

## Conclusion

This meta-analysis underscores lidocaine’s role as a robust prophylactic measure against early postoperative CRBD. IV and intravesical routes substantially lower the occurrence and intensity of moderate-to-severe CRBD in the immediate 2-h window following surgery, though intravesical delivery stands out for its especially strong and enduring impact. Although the broader opioid-sparing benefits fell short of statistical significance, there was a clear dip in morphine use. With its proven effectiveness and reassuring safety record, lidocaine—particularly when administered through intravesical irrigation—merits inclusion as a key element in multimodal approaches to CRBD control among vulnerable surgical cohorts. Future trials should aim to stratify participants by known risk factors (e.g., age, gender) to better define lidocaine’s efficacy in high-risk populations and guide personalized prophylaxis.

## Supplementary Information

Below is the link to the electronic supplementary material.

ESM 1 (DOCX (955 KB) 

## Data Availability

The datasets used and/or analyzed during the current study are available from the corresponding author on reasonable request.

## References

[CR1] Agarwal A, Raza M, Singhal V, Dhiraaj S, Kapoor R, Srivastava A, Gupta D, Singh PK, Pandey CK, Singh U (2005) The efficacy of tolterodine for prevention of catheter-related bladder discomfort: a prospective, randomized, placebo-controlled, double-blind study. Anesth Analg 101:1065–1067 16192522 10.1213/01.ane.0000167775.46192.e9

[CR2] Agarwal A, Dhiraaj S, Singhal V, Kapoor R, Tandon M (2006) Comparison of efficacy of oxybutynin and tolterodine for prevention of catheter related bladder discomfort: a prospective, randomized, placebo-controlled, double-blind study. Br J Anaesth 96:377–380 16415311 10.1093/bja/ael003

[CR3] Agarwal A, Dhiraaj S, Pawar S, Kapoor R, Gupta D, Singh PK (2007) An evaluation of the efficacy of gabapentin for prevention of catheter-related bladder discomfort: a prospective, randomized, placebo-controlled, double-blind study. Anesth Analg 105:1454–1457 17959982 10.1213/01.ane.0000281154.03887.2b

[CR4] Aguilar JoséS, Criado M, De Robertis E (1980) Inhibition by local anesthetics, phentolamine and propranolol of [3H]quinuclydinyl benzylate binding to central muscarinic receptors. Eur J Pharmacol 68:317–326 7202495 10.1016/0014-2999(80)90529-4

[CR5] Bai Y, Wang X, Li X, Pu C, Yuan H, Tang Y, Li J, Wei Q, Han P (2015) Management of catheter-related bladder discomfort in patients who underwent elective surgery. J Endourol 29:640–649 25335575 10.1089/end.2014.0670 PMC4490590

[CR6] Bala I, Bharti N, Chaubey VK, Mandal AK (2012) Efficacy of gabapentin for prevention of postoperative catheter-related bladder discomfort in patients undergoing transurethral resection of bladder tumor. Urology 79:853–857 22309784 10.1016/j.urology.2011.11.050

[CR7] Bozkurt TE, Sahin-Erdemli I (2009) M1 and M3 muscarinic receptors are involved in the release of urinary bladder-derived relaxant factor. Pharmacol Res 59:300–305 19416629 10.1016/j.phrs.2009.01.013

[CR8] Chantrapannik E, Munjupong S, Limprasert N, Jinawong S (2024) Effect of intravenous lidocaine on catheter-related bladder discomfort, postoperative pain and opioid requirement in complex fusion lumbar spinal surgery: a randomized, double blind, controlled trial. BMC Anesthesiol. 10.1186/S12871-024-02789-Y 39528937 10.1186/s12871-024-02789-y PMC11552165

[CR9] Ergenoglu P, Akin S, Yalcin Cok O, Eker E, Kuzgunbay B, Turunc T, Aribogan A (2012) Effect of intraoperative paracetamol on catheter-related bladder discomfort: a prospective, randomized, double-blind study. Curr Ther Res 73:186–194 24653520 10.1016/j.curtheres.2012.08.001 PMC3955106

[CR10] Foo I, Macfarlane AJR, Srivastava D, Bhaskar A, Barker H, Knaggs R, Eipe N, Smith AF (2021) The use of intravenous lidocaine for postoperative pain and recovery: international consensus statement on efficacy and safety. Anaesthesia 76:238–250 33141959 10.1111/anae.15270

[CR11] Hur M, Park S-K, Yoon H-K, Yoo S, Lee H-C, Kim WH, Kim J-T, Ku JH, Bahk J-H (2019) Comparative effectiveness of interventions for managing postoperative catheter-related bladder discomfort: a systematic review and network meta-analysis. J Anesth 33:197–208 30603826 10.1007/s00540-018-2597-2

[CR12] Igawa Y (2000) Discussion: functional role of M1, M2, and M3 muscarinic receptors in overactive bladder. Urology 55:47–49 10767451 10.1016/s0090-4295(99)00493-8

[CR13] Kim DH, Park JY, Yu J, Lee SA, Park S, Hwang JH, Koh GH, Kim YK (2020) Intravenous lidocaine for the prevention of postoperative catheter-related bladder discomfort in male patients undergoing transurethral resection of bladder tumors: A Randomized, Double-Blind, Controlled Trial. Anesth Analg 131(1):220–227. 10.1213/ANE.0000000000004405 10.1213/ANE.0000000000004405 31490257

[CR14] Khan JS, Yousuf M, Victor JC, Sharma A, Siddiqui N (2016) An estimation for an appropriate end time for an intraoperative intravenous lidocaine infusion in bowel surgery: a comparative meta-analysis. J Clin Anesth 28:95–104 26342631 10.1016/j.jclinane.2015.07.007

[CR15] Lauretti GR (2008) Mecanismos envolvidos na analgesia da lidocaína por via venosa. Rev Bras Anestesiol 58:280–286 19378524 10.1590/s0034-70942008000300011

[CR16] Li S, Li P, Wang R, Li H (2022) Different interventions for preventing postoperative catheter-related bladder discomfort: a systematic review and meta-analysis. Eur J Clin Pharmacol 78:897–906 35218404 10.1007/s00228-021-03251-5

[CR17] Li SY, Li H, Ni J, Ma YS (2019) Comparison of intravenous lidocaine and dexmedetomidine infusion for prevention of postoperative catheter-related bladder discomfort: a randomized controlled trial. BMC anesthesiology, 19(1):37. 10.1186/s12871-019-0708-8 10.1186/s12871-019-0708-8 PMC6421662 30885134

[CR18] Lim N, Yoon H (2017) Factors predicting catheter-related bladder discomfort in surgical patients. J Perianesth Nurs 32:400–408 28938975 10.1016/j.jopan.2016.03.012

[CR19] Lin CH, Lu IC, Gau TP, Cheng KI, Chen HL, Hu PY (2024) Preventing postoperative catheter-related bladder discomfort (CRBD) with bladder irrigation using 0.05% lidocaine saline solution: monitoring with analgesia nociception index (ANI) after transurethral surgery. Medicina (b Aires) 60:1405 10.3390/medicina60091405 PMC11433757 39336446

[CR20] Markopoulos T, Katsimperis S, Lazarou L, Tzelves L, Mitsogiannis I, Papatsoris A, Skolarikos A, Varkarakis I (2025) Catheter-related bladder discomfort: insights into pathophysiology, clinical impact, and management. Cureus. 10.7759/cureus.81322 40291191 10.7759/cureus.81322 PMC12034329

[CR21] Marret E, Rolin M, Beaussier M, Bonnet F (2008) Meta-analysis of intravenous lidocaine and postoperative recovery after abdominal surgery. Br J Surg 95:1331–1338 18844267 10.1002/bjs.6375

[CR22] Nam K, Seo J-H, Ryu J-H, Oh A-Y, Lee T, Park H-P, Jeon Y-T, Hwang J-W (2015) Randomized, clinical trial on the preventive effects of butylscopolamine on early postoperative catheter-related bladder discomfort. Surgery 157:396–401 25304838 10.1016/j.surg.2014.05.017

[CR23] Ouzzani M, Hammady H, Fedorowicz Z, Elmagarmid A (2016) Rayyan-a web and mobile app for systematic reviews. Syst Rev 5:1–10 27919275 10.1186/s13643-016-0384-4 PMC5139140

[CR24] Page MJ, McKenzie JE, Bossuyt PM et al (2021) The PRISMA 2020 statement: an updated guideline for reporting systematic reviews. PLoS Med 18:e1003583 33780438 10.1371/journal.pmed.1003583 PMC8007028

[CR25] Ren J, Yu T, Tian Y, Luo G (2023) Comparative effectiveness of interventions for managing urological postoperative catheter-related bladder discomfort: a systematic review and network meta-analysis. BMC Urol. 10.1186/S12894-023-01195-9 36869313 10.1186/s12894-023-01195-9 PMC9985303

[CR26] Schünemann H, Brożek J, Guyatt G, Oxman A. (n.d.) GRADE... - Google Scholar. https://scholar.google.com/scholar?hl=en&q=Sch%C3%BCnemann+H%2C+Bro%C5%BCek+J%2C+Guyatt+G%2C+Oxman+A.+GRADE+handbook.+October+2013.+2013+https%3A%2F%2Fgdt.gradepro.org%2Fapp%2Fhandbook%2Fhandbook.html%23h.svwngs6pm0f2. Accessed 27 Feb 2025

[CR27] Singh A, Kayina CA, Naik N, Ganesh V, Kumar S, Pandey VK, Bora GS, Saini K, Soni S L, Kaloria N, Samra T, Saini V (2023) Transurethral lidocaine (100 mg) bladder irrigation (tuli100) reduces the incidence of catheter related bladder discomfort in transurethral resection of bladder tumors: A randomized, double blind, controlled trial. Int J Urol. official journal of the Japanese Urological Association 30(3):264–270. 10.1111/iju.15100 10.1111/iju.15100 36375083

[CR28] Srivastava VK, Agrawal S, Kadiyala VN, Ahmed M, Sharma S, Kumar R (2015) The efficacy of pregabalin for prevention of catheter-related bladder discomfort: a prospective, randomized, placebo-controlled double-blind study. J Anesth 29:212–216 25200037 10.1007/s00540-014-1911-x

[CR29] Sterne JAC, Savović J, Page MJ et al (2019) RoB 2: a revised tool for assessing risk of bias in randomised trials. BMJ. 10.1136/BMJ.L4898 31462531 10.1136/bmj.l4898

[CR30] Tauzin-Fin P, Sesay M, Svartz L, Krol-Houdek M-C, Maurette P (2007) Sublingual oxybutynin reduces postoperative pain related to indwelling bladder catheter after radical retropubic prostatectomy †. Br J Anaesth 99:572–575 17681969 10.1093/bja/aem232

[CR31] Thorlund K, Engstrøm J, Wetterslev J, JB CTU (2017)‏ Undefined Trial Sequential Analysis (TSA)‏. In: Thorlund K, Engstrøm J, Wetterslev J, Brok J, Imberger G, Gluud‏ Copenhagen C (eds) Copenhagen Trial Unit, Centre for Clinical Intervention Research, 2017‏‏

[CR32] Yamanishi T, Chapple CR, Chess-Williams R (2001) Which muscarinic receptor is important in the bladder? World J Urol 19:299–306 11760777 10.1007/s003450100226

