# Supplementary material for: Evaluation of lidocaine as a dual-route prophylaxis in postoperative catheter-related bladder discomfort: a comprehensive systematic review and meta-analysis with trial sequential analysis and GRADE evaluation
Source: Naunyn Schmiedebergs Arch Pharmacol. 2026 Jan 14;399(6):7863–76. doi: 10.1007/s00210-025-04937-8 (PMC13086742; doi:10.1007/s00210-025-04937-8)
Supplement: Supplementary file 1 — (DOCX (955 KB) [file 210_2025_4937_MOESM1_ESM.docx]

| Study ID | Eligibility criteria | Anesthesia |
| --- | --- | --- |
|  |  |  |
| Chantrapannik et al 2024 | - Eighty male patients aged 20 to 70 years. - Scheduled for complex fusion lumbar spinal surgery at least two levels under general anesthesia. - With American Society of Anesthesiologists physical status, I and II. - Patients with a history of prior urological surgeries, bladder outflow obstruction, overactive bladder, sensory disturbances or bladder issues, spinal surgeries affecting bladder function or pelvic organs, cardiovascular disease, end-stage renal disease, or psychiatric disorders were excluded from the study. | - Group L, 1.5 mg/kg of intravenous lidocaine was administered before inducing anesthesia, followed by a continuous infusion of 2 mg/kg/h during the anesthetic period. - Group C, patients received 0.9% NaCl at the same bolus volume and continuous infusion rate |
| Kim et al 2020 | - Male patients scheduled for elective TURBT under general anesthesia were assessed for eligibility. - Patients were included if they were aged 20–79 years. - With an American Society of Anesthesiologists physical status ≤II. - Patients were excluded if they had heart failure, coronary artery disease, liver cirrhosis, chronic kidney disease, or an allergy to lidocaine. - Patients who had arrhythmias, such as sinus bradycardia, heart block, and atrial fibrillation. | General anesthesia was induced using propofol (2 mg/kg) and rocuronium (0.6 mg/kg). Once the patient was unconscious, a laryngeal mask (LM) was inserted.  Anesthesia was maintained with sevoflurane (2–3 vol%) in a mixture of N2 O 50% and oxygen 50%. |
| Li et al 2019 | - Patients scheduled for underwent elective open abdominal hysterectomy or hysteromyomectomy requiring a urinary bladder catheter were recruited. - Aged 18–60 years - Patients with a history of bladder outflow obstruction, urinary tract infection, OAB, neurogenic bladder, chronic analgesic abuse, severe hepatic or renal disease, arrhythmia, allergy to lidocaine, and morbid obesity were excluded from this trial. - Patients with intra-operative damage to the urinary tract or intestinal tract, massive hemorrhage, or operative time > 6h were removed from this study. | Anesthesia was induced with midazolam 2 mg, sufentanil 3 μg/kg, and propofol 2mg/kg, and intubation was facilitated with rocuronium 0.6mg/kg.  Anesthesia was maintained with sevoflurane and additional sufentanil and rocuronium. |
| Lin et al 2024 | - Inclusion criteria included: Patients who received regular transurethral surgery were inserted left foley catheter for urine drainage or bladder irrigation. - American Society of Anesthesiologists (ASA)class:Ι-Ⅲ - Age:20-70years - Exclusion Criteria: Lidocaine allergy - Cardiac conduction with partial or total block - Patient with progressive hepatic or renal impairment - Patient with class III anti-arrhythmia agent (ex:amiodarone) - Patient with acute porphyria | Anesthesia was induced with 0.5 µg/kg fentanyl, 3 mg/kg thiamylal, and 1 mg/kg propofol.  Anesthesia was maintained with sevoflurane 1~1.3 MAC with an oxygen flow of  0.3 L/min and mixed air of 0.7 L/min |
| Singh et al 2023 | - Adult patients (aged between 20–75 years) with ASA PS(American Society of Anesthesiologists Physical Status) I–II undergoing elective TURBT surgery under general anesthesia were recruited. - Patients who were known cases of arrhythmias, heart block or atrial fibrillation, severe respiratory or heart disease, cirrhosis of the liver or history, urinary catheter in-situ of allergy to lidocaine were excluded. - After pre-anesthetic evaluation and routine investigations as per the institutional protocol patients were kept fasting 8 h for solid food and 2 h for clear fluid | Induced intravenous with fentanyl (2 mcg/kg) followed by 2 mg/kg of propofol and 0.5 mg/kg of atracurium.  Anesthesia was maintained with sevoflurane in a 50:50 mixture of nitrous oxide and oxygen; targeting a MAC of 1 to 1.2. |

**Supplementary table 1.** Eligibility criteria details and anesthesia protocols.

**Supplementary Table. 2** Detailed GRADE evaluation (Lidocaine vs Normal Saline)

| **Outcome** | **Follow up** | **No. of studies** | **No. of patients** | **RR [± 95% CI]** | ***P*- value** | **Heterogeneity assessment**  ***I^2^ [P- value]*** | **Risk of bias** | **Indirectness** | **inconsistency** | **Imprecision** | **Publication bias** | **GRADE Evaluation** |
| --- | --- | --- | --- | --- | --- | --- | --- | --- | --- | --- | --- | --- |
| ***Risk of Moderate to severe CRBD*** | After 0 hours | 5 RCTs | 463 | RR = 0.42, 95% CI: [0.32 to 0.55] | *P* < 0.00001* | *I^2^= 0% [P= 0.82]* | No ^a^ | No | No | No ^c^ | N/A | **⨁⨁⨁⨁ High ^a, c^** |
|  | After 1 hour | 5 RCTs | 463 | RR = 0.42, 95% CI: [0.28 to 0.61] | *P* < 0.00001* | *I^2^= 0% [P=0.70]* | No ^a^ | No | No | No ^c^ | N/A | **⨁⨁⨁⨁ High ^a, c^** |
|  | After 2 hours | 5 RCTs | 463 | RR = 0.33, 95% CI: [0.12 to 0.90] | *P =* 0.03* | *I^2^= 53% [P=0.07]* | No ^a^ | No | Yes ^b^ | Yes ^d^ | N/A | **⨁⨁◯◯ Low ^a, b, d^** |
|  | After 6 hours | 4 RCTs | 383 | RR = 0.30, 95% CI: [0.07 to 0.22] | *P* = 0.09 | *I^2^= 0% [P=0.96]* | No ^a^ | No | No | Yes ^e^ | N/A | **⨁⨁⨁◯ Moderate^a, e^** |

| **CI:** confidence interval; **MD:** mean difference |
| --- |
| **GRADE Working Group grades of evidence** **High certainty:** we are very confident that the true effect lies close to that of the estimate of the effect. **Moderate certainty:** we are moderately confident in the effect estimate: the true effect is likely to be close to the estimate of the effect, but there is a possibility that it is substantially different. **Low certainty:** our confidence in the effect estimate is limited: the true effect may be substantially different from the estimate of the effect. **Very low certainty:** we have very little confidence in the effect estimate: the true effect is likely to be substantially different from the estimate of effect. |

Explanations:

Note: N/A= Although funnel plots revealed a notable degree of symmetry, Egger’s test isn’t applicable because of the small number of included studies.

a. Some concerns in randomization process domain but not rated down to risk of bias.

b. Downgraded one level for inconsistency (I² > 50%, moderate heterogeneity).

c. TSA revealed conclusive results with a sample size exceeding the RIS.

d. Downgraded one level for Imprecision as the TSA revealed inconclusive results with a sample size not exceeding the RIS, moreover owing to the borderline effect as the CI is close to the null.

e. Downgraded for imprecision owing to the wide 95% CI which includes the null “1”, moreover TSA revealed inconclusive results with a sample size not exceeding the RIS.


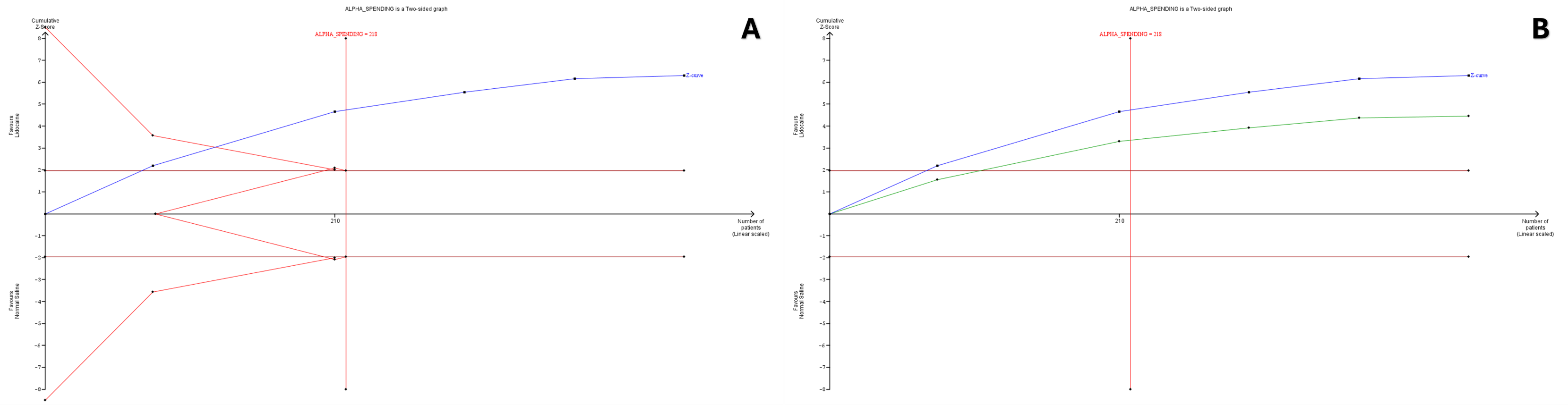


**(Supplementary figure. 1)** Trial sequential analysis on Risk Ratios (RR) of CRBD after 0 hours comparing Lidocaine to Normal saline.

**A)** Alpha spending Adjusted boundaries plot

**B)** Penalized Z-curve plot


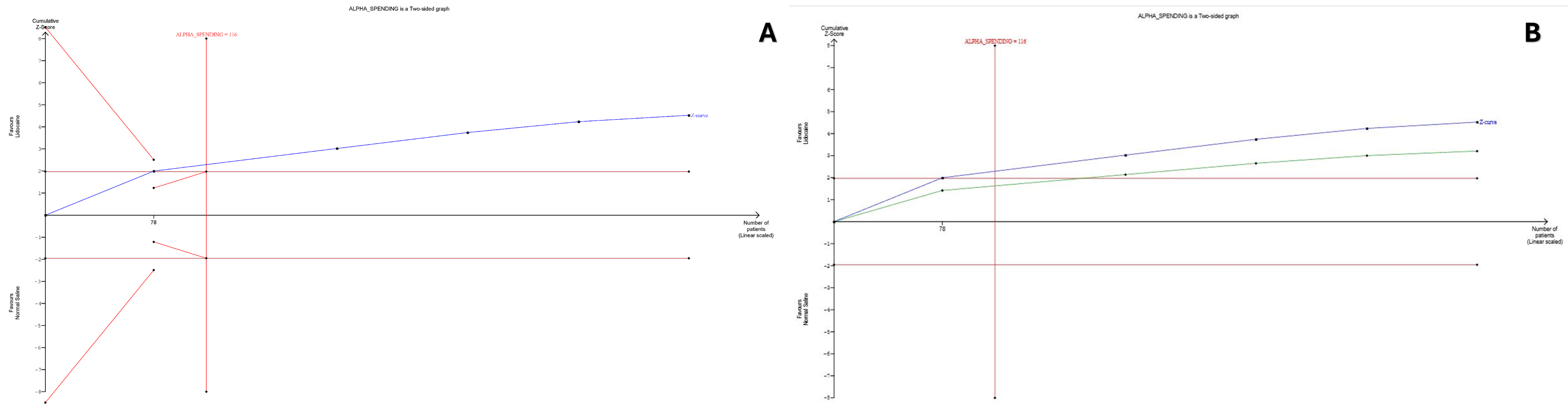


**(Supplementary figure. 2)** Trial sequential analysis on Risk Ratios (RR) of CRBD after 1 hour comparing Lidocaine to Normal saline.

**A)** Alpha spending Adjusted boundaries plot

**B)** Penalized Z-curve plot


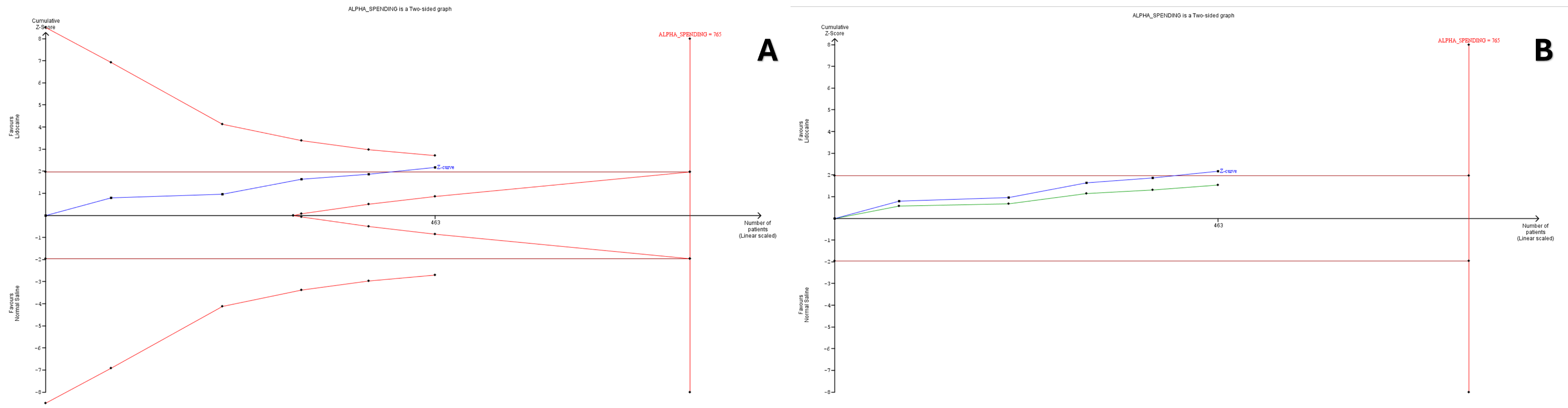


**(Supplementary figure. 3)** Trial sequential analysis on Risk Ratios (RR) of CRBD after 2 hours comparing Lidocaine to Normal saline.

**A)** Alpha spending Adjusted boundaries plot

**B)** Penalized Z-curve plot


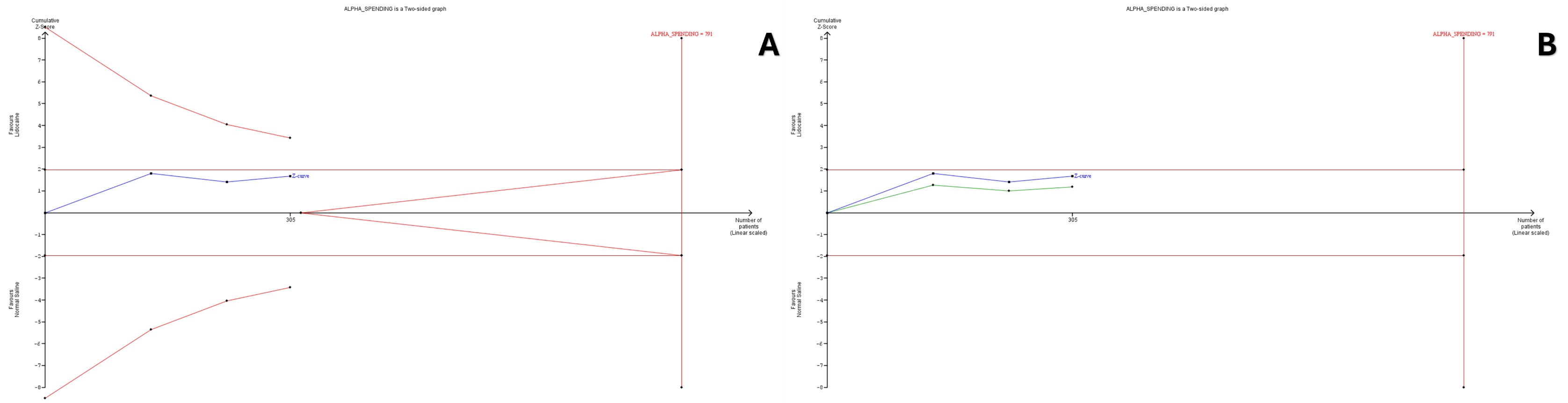


**(Supplementary figure. 4)** Trial sequential analysis on Risk Ratios (RR) of CRBD after 6 hours comparing Lidocaine to Normal saline.

**A)** Alpha spending Adjusted boundaries plot

**B)** Penalized Z-curve plot

**
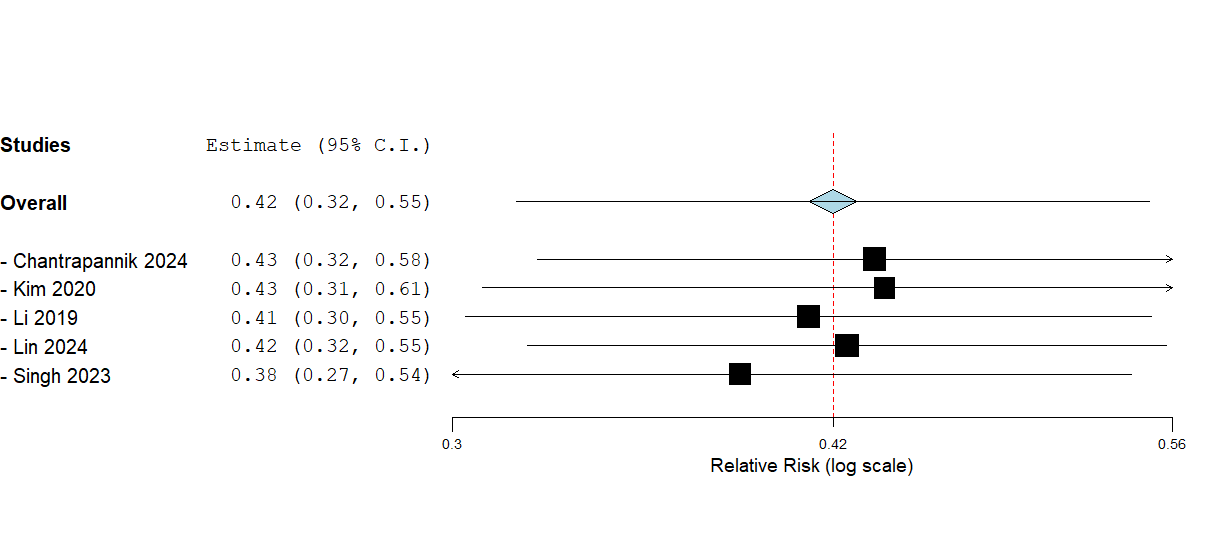
**

**(Supplementary figure. 5)** Leave-one-out-analysis for Risk Ratios (RR) of CRBD after 0 hours comparing Lidocaine to Normal saline.

**
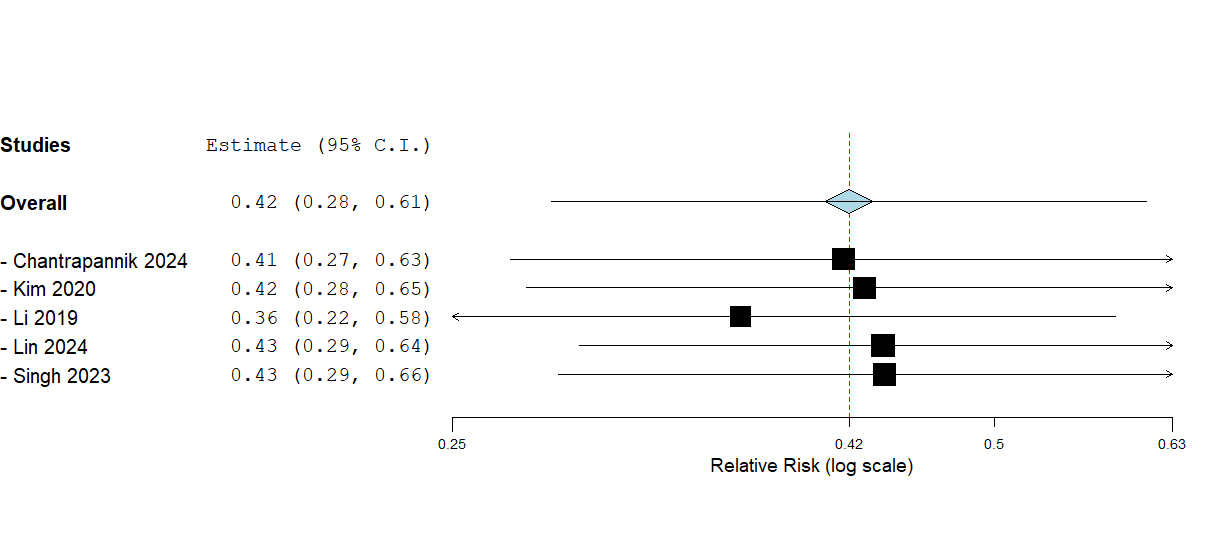
**

**(Supplementary figure. 6)** Leave-one-out-analysis for Risk Ratios (RR)of CRBD after 1 hour comparing Lidocaine to Normal saline.

**
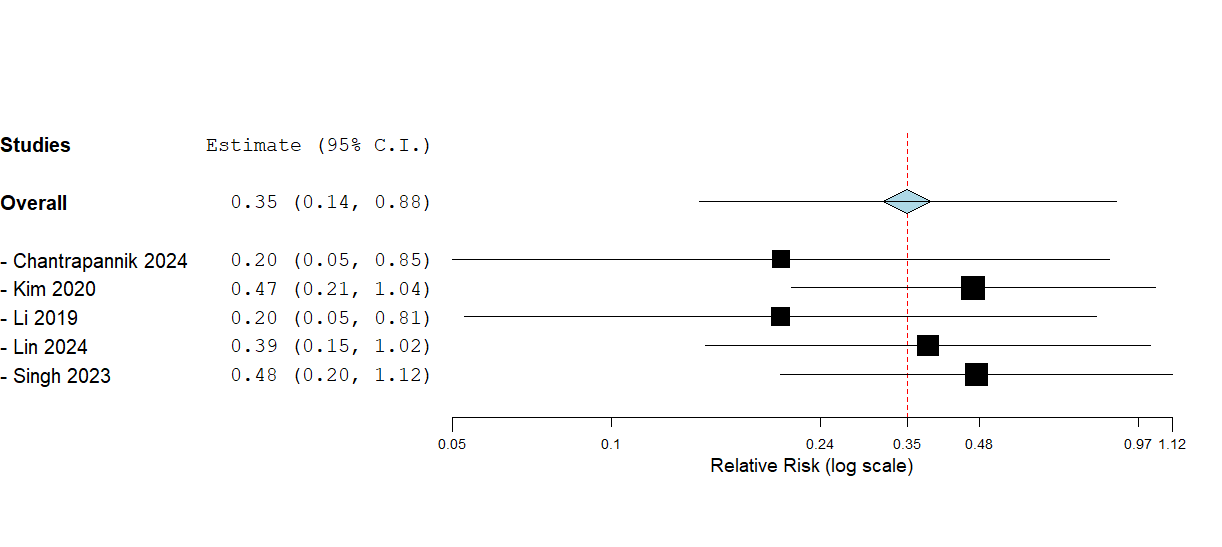
**

**(Supplementary figure. 7)** Leave-one-out-analysis for Risk Ratios (RR) of CRBD after 2 hours comparing Lidocaine to Normal saline.


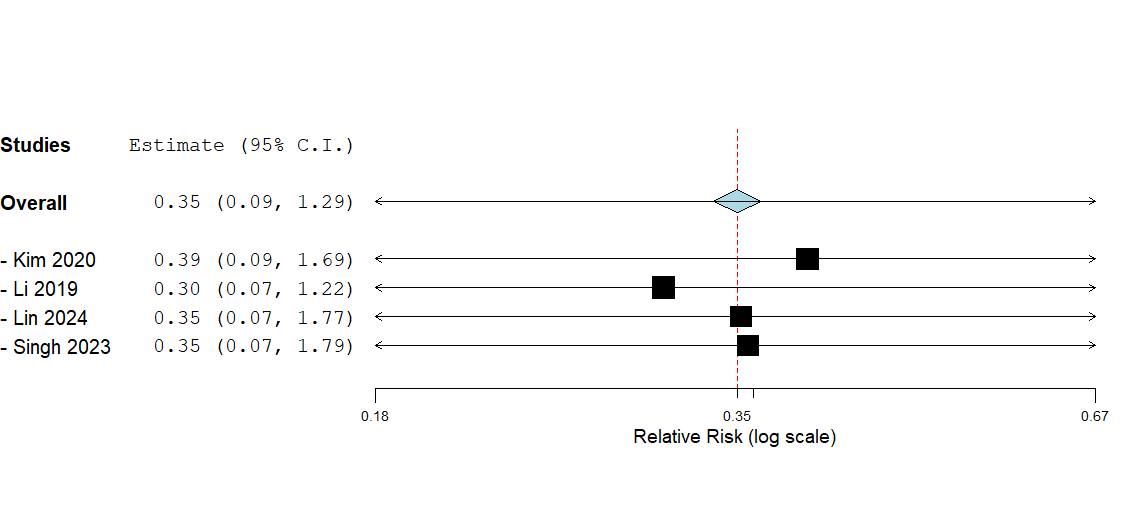


**(Supplementary figure. 8)** Leave-one-out-analysis for Risk Ratios (RR) RR of CRBD after 6 hours comparing Lidocaine to Normal saline.


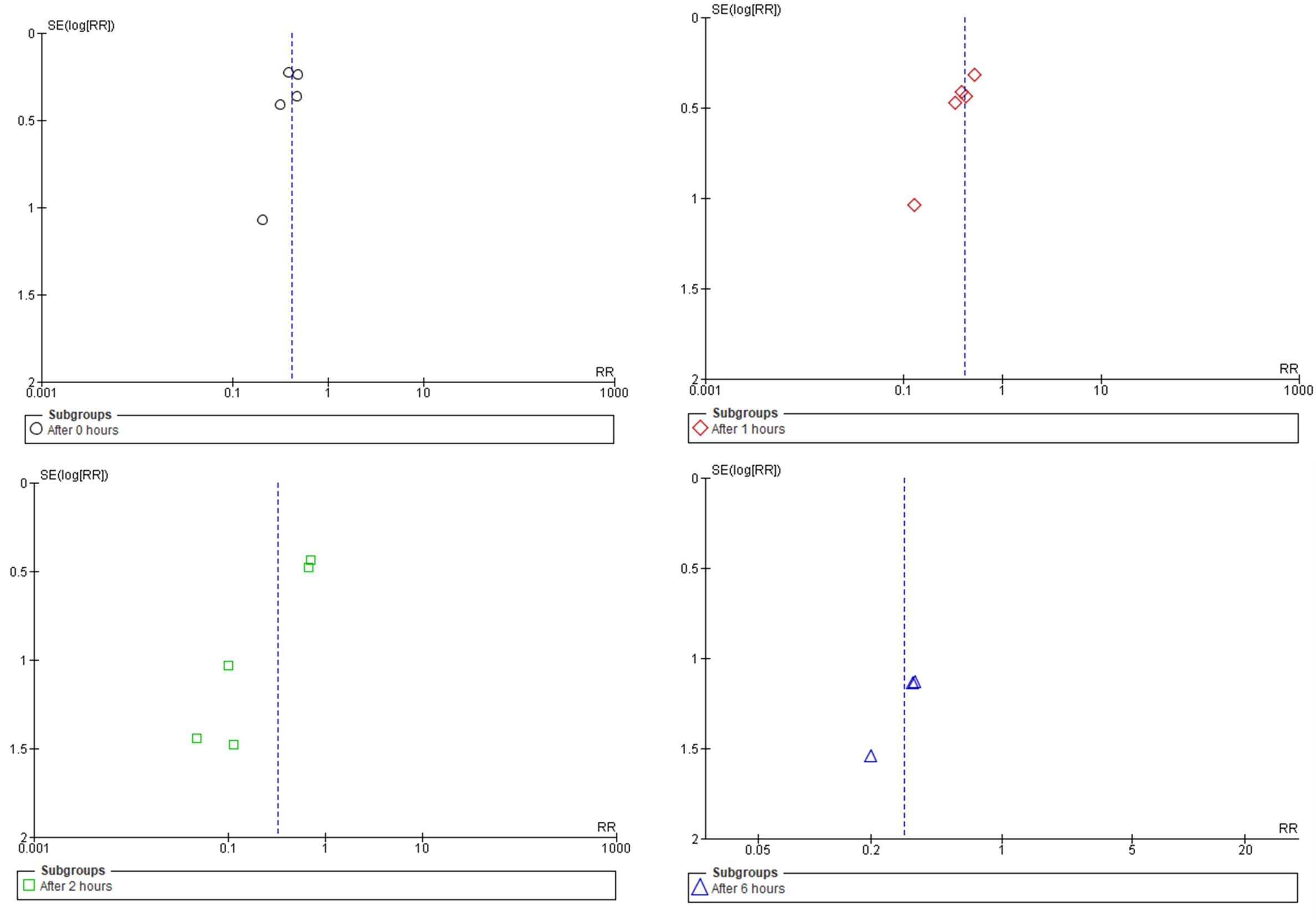


**(Supplementary figure. 9)** Funnel plots for the Incidence of CRBD comparing Lidocaine to Normal saline**.**
